# Development and Characterization of Adeno-Associated Virus-Loaded Coaxial Electrospun Scaffolds for Potential Viral Vector Delivery

**DOI:** 10.3390/polym17101381

**Published:** 2025-05-17

**Authors:** Haiguang Zhang, Bing Zhou, Wei Dong, Yongteng Song, Qingxi Hu, Heng Zhang, Min Yu, Guanglang Zhu, Yudong Sun, Jiaxuan Feng

**Affiliations:** 1 Rapid Manufacturing Engineering Center, School of Mechatronic Engineering and Automation, Shanghai University, Shanghai 200444, China; 2 National Demonstration Center for Experimental Engineering Training Education, Shanghai University, Shanghai 200444, China; 3 Shanghai Key Laboratory of Intelligent Manufacturing and Robotics, Shanghai University, Shanghai 200444, China; 4 Nanjing University, Nanjing 210093, China; 5 School of Medicine, Shanghai Jiaotong University, Shanghai 200240, China

**Keywords:** localized gene delivery, adeno-associated virus, coaxial electrospinning, core–shell structure fiber, tissue engineering

## Abstract

Gene therapy, which treats genetic diseases by fixing defective genes, has gained significant attention. Viral vectors show great potential for gene delivery but face limitations like poor targeting, uncontrolled release, and risks from high-dose delivery which can lower efficiency and trigger immune responses. Loading viral vectors onto tissue engineered scaffolds presents a promising strategy to address these challenges, but their widespread application remains limited due to concerns regarding viral vector bioactivity, scaffold biocompatibility, and the stability of sustained release. An adeno-associated virus (AAV), recognized for its safety, high efficiency, and low immunogenicity, was employed as a model virus. In this study, we developed an electrospun scaffold (AAV/PCL-PEO@Co-ES) by encapsulating the AAV within core–shell fibers composed of polycaprolactone (PCL) and polyethylene oxide (PEO) via coaxial electrospinning. This configuration ensures viral vector protection while enabling controlled and sustained release. The physicochemical characterization results indicated that the scaffold exhibited excellent mechanical properties (tensile strength: 3.22 ± 0.48 MPa) and wettability (WCA: 67.90 ± 8.45°). In vitro release and cell transduction assays demonstrated that the AAV-loaded scaffold effectively controls viral vector release and transduction. Furthermore, both in vitro and in vivo evaluations demonstrated good biocompatibility and efficient viral vector delivery. These findings highlight the potential of the AAV/PCL-PEO@Co-ES scaffold as a safe and effective platform for sustained gene delivery, offering valuable insights for the future design of clinically relevant viral vector delivery systems.

## 1. Introduction

Gene therapy, a revolutionary approach aimed at treating genetic diseases by correcting or replacing defective genes, has attracted widespread attention in recent years [1]. Among various gene delivery strategies such as plasmid DNA and chemical-based systems, viral vectors are particularly promising for treating genetic diseases and promoting tissue regeneration due to their high transfection efficiency, stable gene expression, and ability to target specific cells [2,3,4]. Recent advancements in the clinical application of viral vector-based gene therapy have predominantly focused on ocular diseases, cardiovascular diseases, cancer, and skeletal-related disorders [5,6,7]. However, despite the significant advancements in viral vector-based gene therapy, clinical applications continue to face some substantial challenges, with inherent immunogenicity and difficulties in targeted delivery limiting the success of many clinical trials [8,9,10]. For instance, targeted therapy of heart tissue is especially critical for enhancing treatment efficacy in cardiovascular diseases [11], highlighting the critical need for efficient and targeted delivery systems to enhance the effectiveness of gene therapy [12]. However, current conventional methods for delivering viral vectors, including local and systemic intravenous injections, often require high doses, resulting in systemic toxicity, off-target effects, and low delivery efficiency [13,14]. Hence, the development of controlled delivery methods for viral vectors is critical to improving gene delivery efficiency while reducing off-target effects.

Currently, biomaterials have been widely used as drug delivery carriers and multifunctional platforms enabling localized viral vector delivery and other related factors [15,16]. Various forms of biomaterials, including microspheres, nanoparticles, hydrogels, and electrospun scaffolds, are widely used for viral vector delivery [17,18,19]. Among these, electrospun scaffolds have attracted considerable interest for their ability to encapsulate viral vectors within fibers, effectively mitigating viral immunogenicity and localized delivery while minimizing off-target effects, lowering required viral doses, and prolonging therapeutic efficacy [20,21]. Crucially, the release rate and gene expression duration can be finely tuned by modulating fiber properties such as diameter and porosity, ensuring precise control over treatment outcomes [22]. Moreover, electrospun fiber scaffolds offer a high specific surface area for efficient viral vector loading and a three-dimensional extracellular matrix (ECM)-like network that promotes cell adhesion, proliferation, and differentiation [23]. Various viral loading strategies significantly influence virus performance on electrospun scaffolds, with several approaches reported [19,24,25]. For instance, single-fluid electrospinning (SF-ES) techniques have been used to encapsulate viral vectors within polycaprolactone (PCL) and elastin-like polypeptide (ELP) scaffolds, enabling sustained in vitro release and efficient cell transduction [26]. While this approach ensures a uniformly distributed biomaterial matrix, it has limitations, including potential damage to viral vectors from organic solvents, temperature, or electric fields [19,20,21]. To enhance viral activity within electrospun scaffolds, researchers have explored the incorporation of viral vectors into the core of core–shell fibers composed of materials such as polyester urethane urea (PEUU) and polyester ether urethane urea (PEEUU), allowing for viral release [27]. However, despite these advances in optimizing viral vector delivery systems, their broader application remains limited due to challenges such as reduced viral bioactivity, unstable release profiles, and concerns regarding biocompatibility [28]. To address these limitations and improve the efficiency of viral vector delivery, this study explores a biomaterial-based approach using a well-characterized viral vector and a modified scaffold system tailored for biocompatibility and controlled release. For the selection of viral vectors, this study proposes an adeno-associated virus (AAV) as the model virus due to its low immunogenicity, ability to sustain long-term gene expression, and recognition as one of the most promising gene delivery vectors today [29,30]. Furthermore, previous studies have demonstrated that the types and formulations of biomaterials can induce distinct immune responses, which influence the interactions between viral vectors and immune cells [31]. Specifically, hydrophilic materials, compared to hydrophobic ones, tend to reduce the adhesion of activated monocytes and the production of inflammatory cytokines, thereby mitigating localized immune responses and the formation of foreign body giant cells (FBGC) [32]. PCL, a hydrophobic synthetic polymer with excellent biocompatibility, is widely used in biomedical and pharmaceutical applications due to its suitability for drug delivery systems [33,34]. However, the inherent hydrophobicity of PCL significantly limits its application in drug delivery environments, necessitating hydrophilic modifications. Polyethylene oxide (PEO), a biocompatible, biodegradable, hydrophilic, and water-soluble polymer, is commonly used to enhance the solubility of PCL [35,36].

Hence, building upon the selection of AAV and the identified limitations of conventional scaffold materials, this study introduces a novel viral vector encapsulation system by preparing a core–shell fiber scaffold (AAV/PCL-PEO@Co-ES) via Co-ES technology. The shell layer was designed using a combination of hydrophilic PEO and hydrophobic PCL to regulate release kinetics and improve wettability, while the AAV vectors were encapsulated within the core layer to protect against environmental stress during fabrication and delivery. This configuration enhances viral vector stability, reduces the initial burst release, and promotes localized transduction while minimizing immune responses. The physicochemical properties of the fabricated virus-loaded fiber scaffolds were thoroughly characterized. Furthermore, 293T cells were employed to evaluate the in vitro release profile and gene transduction efficiency via mCherry expression, a red fluorescent protein used as a reporter gene. In vivo biocompatibility and delivery performance were assessed through subcutaneous implantation in Sprague Dawley (SD) rats.

## 2. Materials and Methods

### 2.1. Materials

PCL (Mw 80 kDa) was purchased from Macklin Chemicals Co., Ltd. (Shanghai, China). PEO (Mw 600 kDa) was obtained from Sigma‒Aldrich Trading Co., Ltd. (Shanghai, China). The solvents dichloromethane (DCM), N, N dimethylformamide (DMF), and phosphate-buffered saline (PBS, pH 7.2) were purchased from Sinopharm Chemical Reagent Co., Ltd. (Shanghai, China). AAV serotype 9 (AAV9-CMV-mCherry, 10^13^ GC/mL) was purchased from PackGene Biotech (Guangzhou, Guangdong, China).

### 2.2. Fabrication of AAV-Loaded Scaffolds

**Figure 1:**
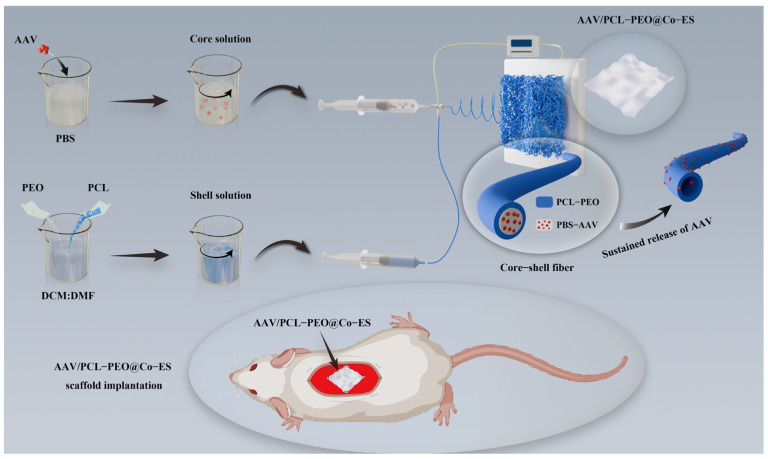
Schematic diagram of the manufacturing process for the AAV/PCL-PEO@Co-ES scaffold.

Figure 1 presents a schematic diagram of the manufacturing process for AAV-loaded fibers, exhibiting the production of core–shell fiber scaffolds (AAV/PCL-PEO@Co-ES) using Co-ES technology. For the preparation of AAV/PCL-PEO@Co-ES, AAV viral particles were mixed with PBS for the core solution, while the shell solution was prepared by dissolving PCL and PEO (9:1 *w*/*w*) in DCM:DMF (7:3 *v*/*v*) to create a 12% (*w*/*v*) PCL-PEO solution. The core and shell solutions were delivered separately into the inner (22G) and outer (17G) needles of a Co-ES needle using two syringe pumps, with flow rates of 0.2 mL/h (core) and 1.5 mL/h (shell), respectively. Furthermore, the aforementioned polymers were mixed with AAV and SF-ES technology was employed to fabricate the conventional fiber scaffolds (AAV/PCL-PEO@SF-ES), which facilitated the comparison of controlled release and transduction efficiency of the viral vectors. For the preparation of AAV/PCL-PEO@SF-ES, the AAV viral particles were dispersed in the PCL-PEO solution, which had the same solution ratio as the shell solution described above, and delivered through a 22G electrospinning needle at a flow rate of 1.5 mL/h using a single syringe pump. Subsequently, two types of scaffolds were prepared using an electrospinning machine (E05-001, Foshan Lepton Precision Measurement and Control Technology Co., Ltd., Foshan, China) under the following conditions: a high voltage of 14 kV, a collection distance of 17 cm, and a working temperature of 25 °C. After 7 h of electrospinning, the AAV/PCL-PEO@Co-ES and AAV/PCL-PEO@SF-ES scaffolds were prepared and freeze-dried in a vacuum dryer (vacuum: 8.0 Pa; temperature: −78 °C) for 8 h. Finally, the resulting AAV-loaded fiber scaffolds were cut into 2 cm × 2 cm × 100 μm samples (~10^10^ GC/sample) for subsequent in vitro/vivo characterization. The theoretical AAV loading per sample was estimated based on the total amount of virus incorporated into the full AAV-loaded scaffold, which was calculated by multiplying the AAV concentration in the electrospinning solution (GC/mL), the flow rate (mL/h), and the total electrospinning time (h). The viral load in each individual sample was then estimated according to its area proportion relative to the entire scaffold. For mechanical testing, scaffolds were also cut into 40 mm × 10 mm strips, as needed for specific tests.

### 2.3. Characterization of the Physicochemical Properties of the Scaffold

#### 2.3.1. Micromorphology Characterization

The surface morphology of the AAV/PCL-PEO@SF-ES and AAV/PCL-PEO@Co-ES scaffolds was characterized using tungsten filament scanning electron microscopy (SEM, SU-1500, Hitachi, Tokyo, Japan). All dried scaffolds were gold-coated under a vacuum before SEM observation. The fiber diameters in the SEM images were measured from the SEM images using Nano Measurer 1.2 software and the average diameter and distribution frequency of 100 fibers were calculated. To further validate the microstructure of the two fiber scaffolds, transmission electron microscopy (TEM, JEM-2010F, JEOL, Beijing, China) was used to image the fibers collected on carbon-coated copper grids, enabling the observation of whether the scaffold fibers exhibited a clear core–shell structure.

#### 2.3.2. Fourier Transform Infrared Spectroscopy Analysis

The chemical composition of the biomaterials PCL, PEO, and the PCL-PEO@Co-ES scaffold without AAV was analyzed using a Fourier transform infrared spectrometer (FTIR, AVATAR370, Nicolet, Wayzata, USA). The FTIR spectra were recorded over a wavenumber range of 4000–400 cm^−1^ with a resolution of 1 cm^−1^.

#### 2.3.3. Mechanical Properties Tests

The mechanical properties of PCL@SF-ES, PCL@Co-ES, PCL-PEO@SF-ES, and PCL-PEO@Co-ES scaffolds (without AAV) were evaluated using a universal materials testing machine (WDW-1, Songdun, Chaozhou, China). PCL@SF-ES and PCL@Co-ES scaffolds were fabricated from a pure PCL solution (12 *w*/*v*% in a 7:3 ratio of DCM:DMF) and used as the control group. Following the previous protocol [34,37], scaffolds were cut into 40 mm × 10 mm strips and their thickness was measured using a digital micrometer. The samples were then clamped at both ends of the testing machine and stretched vertically at a strain rate of 5 mm/min until failure and the stress–strain curves were obtained. The elastic modulus was then calculated for each sample (*n* = 3).

Considering that the hydrophilic PEO rapidly dissolves in aqueous solutions, which can accelerate fiber degradation even within hours, it is essential to evaluate the mechanical properties of the fibers after immersion in an aqueous environment. Therefore, the above-mentioned scaffolds were immersed in PBS at room temperature for 4 h. After immersion, the stress–strain curves of the wet scaffolds were measured using the same method (*n* = 3).

#### 2.3.4. Surface Wettability Analysis

The wettability of the PCL@SF-ES, PCL@Co-ES, PCL-PEO@SF-ES, and PCL-PEO@Co-ES scaffolds was evaluated using a contact angle measuring instrument (JC2000D1, Powereach, Shanghai, China). As described in the former protocols [38,39], 4 μL of deionized water was dropped on the surface of each scaffold and real-time images were captured to directly measure the water contact angle (WCA) (*n* = 5).

#### 2.3.5. Degradation Performance Tests

The degradation performance of the PCL@SF-ES, PCL@Co-ES, PCL-PEO@SF-ES, and PCL-PEO@Co-ES scaffolds was evaluated through the gravimetric analysis method. Before testing, the scaffolds (*n* = 3) were dried and weighed to determine their initial weight (*W*_a_). The scaffolds were then incubated in PBS solution at 37 °C and retrieved at predetermined time points (1, 3, 6, 12, 24, 48, and 72 h). After drying the scaffolds to a constant weight at 37 °C, the final weight (*W*_b_) was recorded. The mass loss rate of the scaffolds was calculated using the following equation [34,38]:

Mass loss = (*W*_a_ − *W*_b_)/*W*_a_ × 100%, (1)

### 2.4. Cell Characterization of the Scaffold

#### 2.4.1. Cell Culture

Human umbilical vein endothelial cells (HUVECs) were obtained from Shanghai ZhongqiaoXinzhou Biotechnology Co., Ltd., Shanghai, China. HUVECs and 293T cells (ATCC, Manassas, VA, USA) were cultured in complete medium at 37 °C in a humidified incubator with 5% CO_2_. The complete medium was prepared by supplementing high-glucose Dulbecco’s modified Eagle’s medium (DMEM, Servicebio, Wuhan, China) with 10% fetal bovine serum (FBS, Thermo Fisher Scientific, Waltham, MA, USA) and 1% penicillin (100 units/mL; Thermo Fisher Scientific, USA) (100 units/mL). During the culture process, cells were passaged and separated using 0.25% trypsin–EDTA when they reached approximately 80% confluence.

#### 2.4.2. Cell Adhesion Assay

To assess the effects of the PCL@SF-ES, PCL@Co-ES, PCL-PEO@SF-ES, and PCL-PEO@Co-ES scaffolds on the growth and adhesion of HUVECs and 293T cells, cells were seeded at a density of 5.0 × 10^5^ cells/mL onto the surface of the sterilized scaffolds. After 4 h of incubation, allowing the cells to adhere to the scaffolds, the fresh complete medium was added to fully immerse the scaffolds. Cell viability was evaluated on days 1, 3, and 5 using a calcein AM/PI double staining kit (Maokang Biotechnology Co., Inc., Shanghai, China). Live and dead cells on the scaffold surface were observed under an inverted fluorescence microscope (LHM100CB-1, Nikon, Tokyo, Japan).

#### 2.4.3. Cell Proliferation Assay

Cell proliferation was quantitatively analyzed using the cell counting kit-8 (CCK-8) reagent (Key-GEN Biotech Co., Ltd., Nanjing, China) [34,39]. The sterilized scaffolds were soaked in the complete medium for 48 h to prepare scaffold leaching solution. HUVECs and 293T cells were seeded at a density of 1 × 10^5^ cells/mL into 96-well plates. After 4 h of incubation to allow for complete cell attachment, the original medium was removed and 100 μL of scaffold leaching solution and fresh medium (the positive control group) were added to each well. After 1, 3, and 5 days of culture, 10 μL of CCK-8 reagent was added to each well, followed by 1 h of incubation. The absorbance at 450 nm was measured using a microplate reader (Infinite 200Pro, Tecan Group Ltd., Männedorf, Switzerland).

### 2.5. In Vitro AAV Release and Cell Transduction Assay

#### 2.5.1. AAV Release in the Scaffold Leaching Solution

The release kinetics of the AAV from the AAV/PCL-PEO@SF-ES and AAV/PCL-PEO@Co-ES scaffolds were analyzed by quantitatively the viral particles in the scaffold leaching solution. Two groups of scaffolds (~10^10^ GC/sample, *n* = 3) were placed in 12-well plates and 1 mL of culture medium was added to each well. The plates were incubated in a culture incubator, and at predetermined time points (1, 2, 4, 8, 12, 24, 48, and 72 h) the scaffold leaching solution was collected and replaced with fresh culture medium. The AAV DNA in the leaching solution was extracted using the SteadyPure Virus DNA/RNA Extraction kit (Accurate Biotechnology (Hunan) Co., Ltd., Changsha, China) and quantified with a Nanodrop 2000 spectrophotometer (Thermo, USA). The primers for AAV DNA (Forward: 5′-GGAACCCCTAGTGATGGAGTT-3′, Reverse: 5′-CGGCCTCAGTGAGCGA-3′) were custom-designed by Sangon Biotech (Shanghai, China). The extracted DNA was then mixed with BrightCycle Universal SYBR Green qPCR Mix with UDG (ABclonal Biotechnology Co., Ltd., Wuhan, China) and the primers and then subjected to qPCR analysis using the LightCycler 480 Instrument (Roche, Basel, Switzerland). The percentage of AAV released over time was calculated by dividing the quantified AAV release amount by the theoretical initial AAV load on each scaffold.

#### 2.5.2. Cell Transduction

To evaluate the transduction efficiency of the AAV/PCL-PEO@SF-ES and AAV/PCL-PEO@Co-ES scaffolds, the scaffold leaching solution collected at the specified time points were used to transduce 293T cells. In a 12-well plate, 293T cells (passages 15–20) were seeded at a density of 1 × 10^5^ cells/well and incubated overnight with the collected scaffold leaching solution, which were then replaced with fresh medium. After 7 days of culture, the cells were stained with DAPI (Shanghai Maokang Biotechnology Co., Ltd., Shanghai, China) to visualize cell nuclei. Fluorescence imaging of the 293T cells was performed using an inverted fluorescence microscope (LHM100CB-1, Nikon, Japan).

### 2.6. In Vivo Animal Experiment

#### 2.6.1. AAV-Loaded Scaffold Implantation

To further assess the in vivo release kinetics, transduction efficiency, and biocompatibility of the AAV/PCL-PEO@SF-ES and AAV/PCL-PEO@Co-ES scaffolds, subcutaneous implantation experiments were performed using 21 male SD rats (180–200 g) obtained from Shanghai Slake Experimental Animal Co., Ltd. Briefly, after anesthetizing the rats, a 25 mm × 25 mm subcutaneous area on the dorsal skin was surgically exposed and AAV-loaded scaffolds (~10^10^ GC/sample) were secured to the subcutaneous tissue with surgical sutures. The surgical incisions were subsequently sutured. The experiment included three groups: AAV/PCL-PEO@SF-ES, AAV/PCL-PEO@Co-ES, and a control group without scaffold implantation. Scaffolds were removed at predetermined time points (24 h, 48 h, and 2 weeks; *n* = 3), and subcutaneous tissues from all groups were harvested 2 weeks post-surgery for analysis. All animal experiments were approved by the Ethics Committee of the Institute of Neuroscience and Intelligent Technology, Chinese Academy of Sciences (Approval No. NA-055-2023-R1).

#### 2.6.2. Histological Examinations

For histological analysis, tissues were fixed in 4% polyoxymethylene for 24 h, embedded in paraffin, and sectioned into thin slices. The tissue sections were stained with Hematoxylin and eosin (H&E) and Masson’s trichrome (Masson, Richmond, BC, Canada) to evaluate tissue growth and inflammation. For tissue transduction analysis, the tissues were fixed in 4% polyoxymethylene for 10 min, rapidly frozen, sectioned at −20 °C, and analyzed under a fluorescence microscope to observe transduction within the tissue.

### 2.7. Statistical Analysis

All experimental results were presented as the mean ± standard deviation (SD). Statistical analyses were performed using Origin 2020 (9.8.0.200), ImageJ (1.54f), and GraphPad Prism (9.5). Group differences were assessed using one-way analysis of variance (ANOVA), with significance denoted as * *p* < 0.05 and ** *p* < 0.01.

## 3. Results and Discussion

### 3.1. Characterization of the Physicochemical Properties of the Scaffold

#### 3.1.1. Micromorphology Characterization

**Figure 2:**
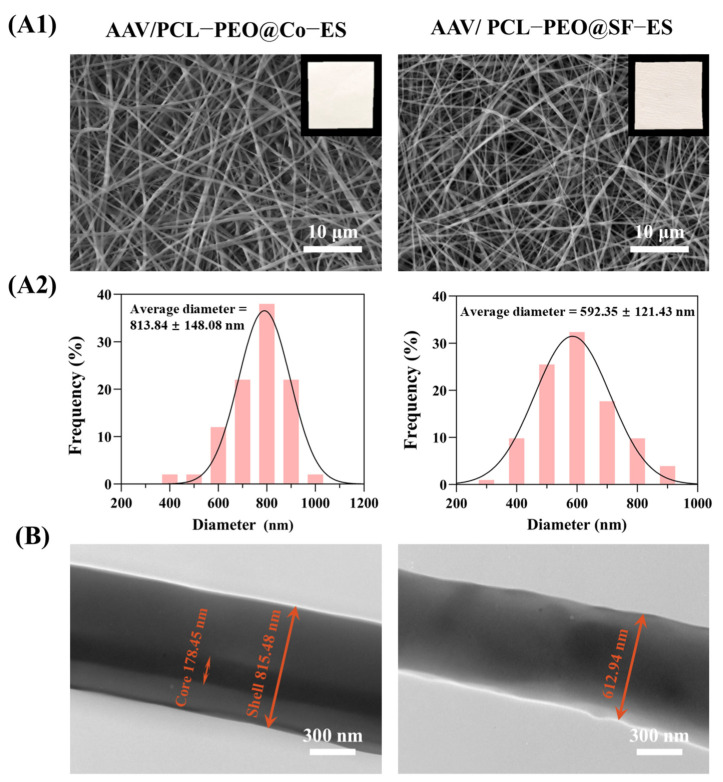
(**A1**,**A2**) Macroscopic and microscopic (SEM) images of AAV/PCL-PEO@Co-ES and AAV/PCL-PEO@SF-ES scaffolds, along with the statistical results of fiber diameter distribution for each group. (**B**) TEM images of AAV/PCL-PEO@Co-ES and AAV/PCL-PEO@SF-ES scaffolds.

The microstructure of the AAV/PCL-PEO@SF-ES and AAV/PCL-PEO@Co-ES scaffolds was characterized using SEM and TEM. Figure 2(A1) displays the macroscopic images (upper right) and surface microstructure of the two scaffolds. Macroscopically, all scaffolds exhibited smooth, uniform surfaces. SEM images revealed continuous, intact fibers without visible beaded structures. The randomly distributed fibers formed a porous structure favorable for cell adhesion and proliferation. Figure 2(A2) presents the statistical analysis of fiber diameters for the two groups of scaffolds. The average fiber diameters of AAV/PCL-PEO@SF-ES and AAV/PCL-PEO@Co-ES scaffolds were 592.35 ± 121.43 nm and 813.84 ± 148.08 nm, respectively. Figure 2B shows the fiber TEM images of the two scaffolds. The AAV/PCL-PEO@Co-ES fibers demonstrated a distinct core–shell structure, with an outer shell approximately 815.48 nm thick and an inner core about 178.45 nm in diameter, indicating great interfacial bonding. In contrast, the AAV/PCL-PEO@SF-ES fibers had a diameter of approximately 612.94 nm and displayed a characteristic solid-core structure.

#### 3.1.2. FTIR Analysis

**Figure 3:**
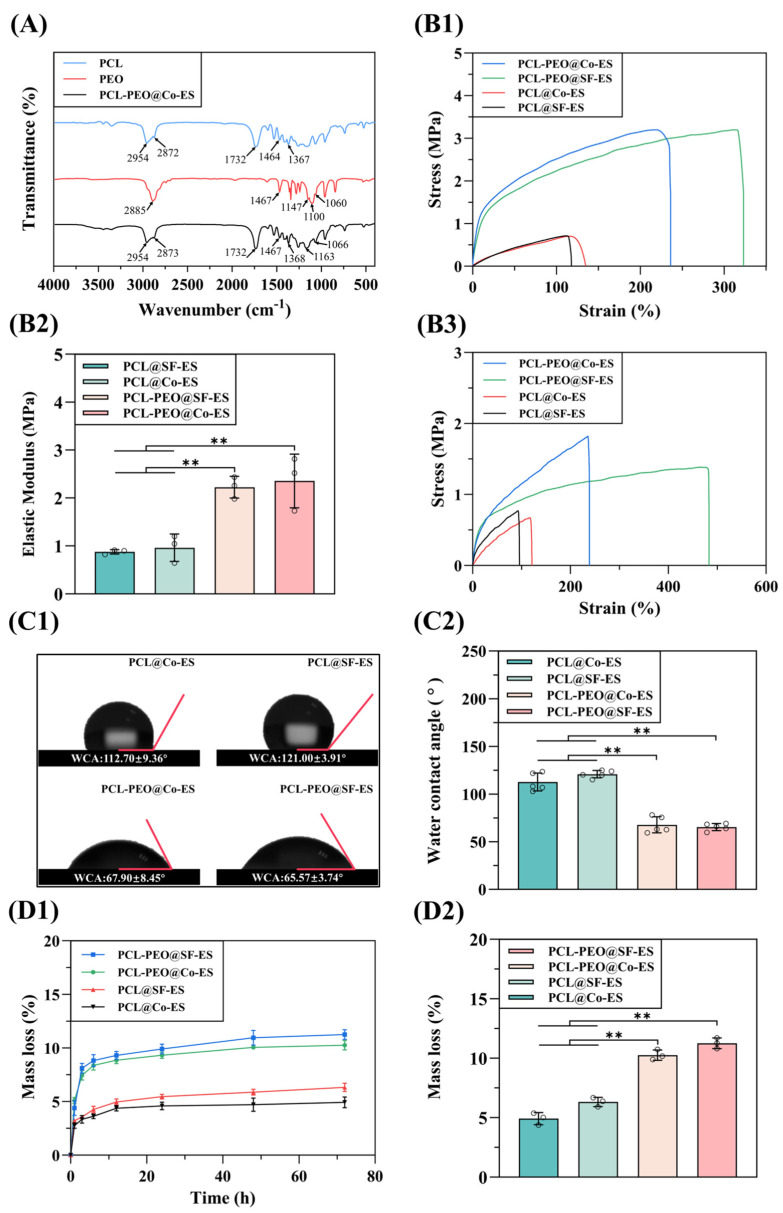
(**A**) FTIR spectra of PCL, PEO, and PCL-PEO@Co-ES. (**B1**–**B3**) The stress–strain curves of dry scaffolds, elastic modulus, and stress–strain curves of wet scaffolds at room temperature for the PCL@SF-ES, PCL@Co-ES, PCL-PEO@SF-ES, and PCL-PEO@Co-ES. (**C1**,**C2**) The WCA diagram and corresponding statistical results of PCL@SF-ES, PCL@Co-ES, PCL-PEO@SF-ES, and PCL-PEO@Co-ES. (**D1**,**D2**) Mass loss rate curves and mass loss rate on day 3 of PCL@SF-ES, PCL@Co-ES, PCL-PEO@SF-ES, and PCL-PEO@Co-ES. The error bars represent standard deviations ((**B1**–**B3**): *n* = 3; (**C1**,**C2**): *n* = 5; (**D1**,**D2**): *n* = 3), with significance levels of ** *p* < 0.01 indicating statistically meaningful significance. Circles in the figure represent individual data points used for statistical analysis.

Figure 3A presents the FTIR spectra of PCL, PEO, and PCL-PEO@Co-ES scaffolds. The PCL spectrum exhibited characteristic peaks at 2954 and 2872 cm^−1^ (CH_2_ symmetric vibration), 1732 cm^−1^ (C=O stretching vibration), and 1464 and 1370 cm^−1^ (C-H bending vibration) [40]. In the PEO spectrum, absorption peaks were observed at 2885 cm^−1^ (C-H stretching vibration), 1467 cm^−1^ (CH_2_ scissoring vibration), and three peaks at 1147, 1100, and 1060 cm^−1^ (C-O-C stretching vibrations) [41]. For the PCL-PEO@Co-ES scaffold spectrum, the typical peaks of PCL (1, 2, and 3) and PEO (4, 5, and 6) were observed separately, indicating that the chemical composition of the materials in the scaffold remained stable and unchanged after mixing.

#### 3.1.3. Mechanical Properties Evaluation

Evaluating the mechanical properties of biological scaffolds is crucial for their application in tissue engineering. As shown in Figure 3(B1,B2), the tensile strengths of the dry PCL@Co-ES, PCL@SF-ES, PCL-PEO@Co-ES, and PCL-PEO@SF-ES scaffolds were 0.63 ± 0.23 MPa, 0.61 ± 0.52 MPa, 3.22 ± 0.48 MPa, and 3.16 ± 0.58 MPa, respectively. The corresponding elongation at break was 148.50 ± 14.97%, 98.48 ± 15.82%, 263.70 ± 49.58%, and 364.40 ± 29.08%, respectively. Furthermore, the corresponding elastic moduli were 0.96 ± 0.28 MPa, 0.87 ± 0.04 MPa, 2.35 ± 0.56 MPa, and 2.22 ± 0.22 MPa, respectively. The results indicate that the addition of PEO to the PCL-PEO scaffolds significantly improved their tensile strength, elongation at break, and elastic modulus compared to pure PCL scaffolds. These findings are consistent with previous studies and can be attributed to the synergistic effects of PCL-PEO blending at the nanoscale [42]. Moreover, the higher elongation at break observed in PCL-PEO@SF-ES scaffolds compared to PCL-PEO@Co-ES scaffolds may be attributed to the smaller diameter and fewer defects in the PCL-PEO@SF-ES fibers, which contribute to the improved mechanical properties of the scaffold [43].

The mechanical properties of the scaffolds were evaluated under wet conditions after immersion in PBS for 4 h. As shown in Figure 3(B3), the tensile strengths of the wet PCL@Co-ES, PCL@SF-ES, PCL-PEO@Co-ES, and PCL-PEO@SF-ES scaffolds were 0.58 ± 0.04 MPa, 0.59 ± 0.05 MPa, 1.88 ± 0.10 MPa, and 1.22 ± 0.10 MPa, respectively. The corresponding elongation at break was 84.62 ± 24.24%, 96.17 ± 31.74%, 225.90 ± 75.1%, and 406.6 ± 98.98%, respectively. In addition, the elastic moduli were 0.96 ± 0.28 MPa, 0.75 ± 0.12 MPa, 1.28 ± 0.30 MPa, and 0.97 ± 0.11 MPa, respectively.

The results demonstrate that the tensile strength and elastic modulus of the wet scaffolds were significantly reduced compared to their dry counterparts. The mechanical properties of the PCL@SF-ES and PCL@Co-ES scaffolds exhibited only slight reductions after immersion, likely due to the hydrophobic nature and relatively slow degradation rate of PCL. In contrast, the PCL-PEO@SF-ES and PCL-PEO@Co-ES scaffolds showed more pronounced decreases in mechanical performance, primarily attributed to the hydrophilic nature of PEO, which rapidly dissolves in aqueous conditions and accelerates fiber degradation. Nevertheless, despite this reduction, the PCL-PEO@SF-ES and PCL-PEO@Co-ES scaffolds still demonstrated superior mechanical properties after PBS immersion compared to the pure PCL scaffolds.

#### 3.1.4. Surface Wettability Analysis

The wettability of the scaffold surface is a critical factor influencing cell adhesion, proliferation, and drug release [44,45]. The scaffold wettability was assessed by measuring the WCA, where a WCA greater than 90° exhibits hydrophobicity and a WCA less than 90° indicates hydrophilicity. The WCA test results for each scaffold are shown in Figure 3(C1,C2). The WCA values of the PCL@Co-ES, PCL@SF-ES, PCL-PEO@Co-ES, and PCL-PEO@SF-ES scaffolds were 112.70 ± 9.36°, 121.00 ± 3.91°, 67.90 ± 8.45°, and 65.57 ± 3.74°, respectively. Experimental results indicate that the PCL@Co-ES and PCL@SF-ES scaffolds fabricated from pure PCL possess limited hydrophilicity. The addition of PEO markedly improves the hydrophilicity of PCL-PEO@Co-ES and PCL-PEO@SF-ES scaffolds, thereby facilitating cell adhesion and proliferation.

#### 3.1.5. Degradation Performance Tests

The degradability of biological scaffolds is a crucial characteristic in tissue engineering applications, enabling the controlled release of bioactive molecules and promoting tissue regeneration [46]. Figure 3(D1,D2) illustrates the mass loss rate of PCL@Co-ES, PCL@SF-ES, PCL-PEO@Co-ES, and PCL-PEO@SF-ES scaffolds over time, along with their mass loss rates after 3 days of degradation. The results indicate that all groups of scaffolds are degradable, with hydrolytic stability dependent on their composition. Specifically, after 3 days, the mass loss rates of PCL@Co-ES and PCL@SF-ES scaffolds were only 4.91 ± 0.50% and 6.31 ± 0.38%, respectively, reflecting the superior water stability of hydrophobic PCL. Consistent with reports suggesting that PCL degrades completely in tissue fluid within 1–3 years [47], these scaffolds exhibited no significant mass loss. In contrast, PCL-PEO@Co-ES and PCL-PEO@SF-ES scaffolds containing PEO showed higher mass loss during the first 24 h, followed by slower degradation. After 3 days, their mass loss rates reached 10.26 ± 0.43% and 11.26 ± 0.45%, respectively, attributed to the hydrophilicity of PEO, which dissolves easily in water. Therefore, the PCL-PEO@Co-ES and PCL-PEO@SF-ES scaffolds can release active substances through short-term mass loss.

### 3.2. Cell Characterization of Scaffold

#### 3.2.1. Cell Adhesion Analysis

**Figure 4:**
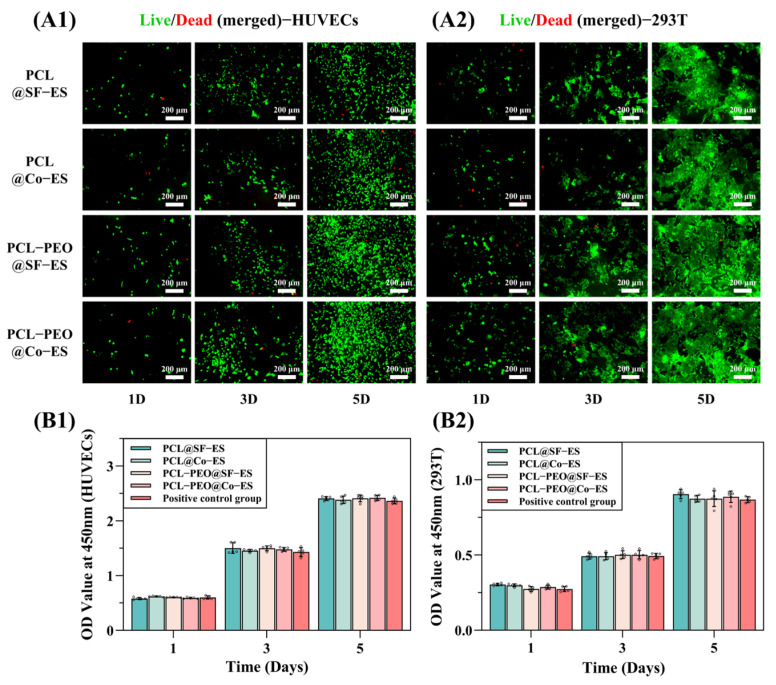
(**A1**,**A2**) Images of live/dead cell staining images (merged) of HUVECs (**A1**) and 293T (**A2**) after culture on PCL@SF-ES, PCL@Co-ES, PCL-PEO@SF-ES, and PCL-PEO@Co-ES on days 1, 3, and 5. (**B1**,**B2**) CCK-8 assay results for HUVECs (**B1**) and 293T (**B2**) after 1, 3, and 5 days of culture. The error bars depict the standard deviation (*n* = 5). Circles in the figure represent individual data points used for statistical analysis.

Biocompatibility is one of the most important indicators for assessing the suitability of biological scaffolds in tissue engineering applications [48]. To assess the biocompatibility of the prepared scaffolds, HUVECs and 293T cells were seeded onto PCL@SF-ES, PCL@Co-ES, PCL-PEO@SF-ES, and PCL-PEO@Co-ES scaffolds. Cell adhesion and growth were observed after 1, 3, and 5 days of culture. Figure 4(A1,A2) shows the live/dead fluorescence images of HUVECs and 293T cells on each group of scaffolds, where green represents live cells and red represents dead cells. The fluorescence images reveal a gradual increase in cell density on the surface of all scaffolds over time. On day 1 of culture, most seeded cells survived and adhered well to the scaffold surface, with few dead cells. By days 3 and 5, both cell types exhibited excellent spreading and proliferation on all scaffold groups, indicating that the scaffolds had good cell adhesion properties. Furthermore, the cell density on PCL-PEO@SF-ES and PCL-PEO@Co-ES scaffolds was slightly higher than on pure PCL scaffolds. This aligns with the wettability results, suggesting that the addition of hydrophilic PEO in the scaffolds enhances cell adhesion and proliferation.

#### 3.2.2. Cell Proliferation Assay

To further quantify the effect of the prepared scaffolds on cell proliferation, a CCK-8 assay was performed to evaluate the impact of the scaffolds’ leaching solution on the proliferation of HUVECs and 293T cells. After culturing the two cell types with the leaching solution from each group of scaffolds for 1, 3, and 5 days, the CCK-8 results (Figure 4(B1,B2)) showed a time-dependent increase in OD values, with consistent growth trends across all groups. Compared to the positive control group cultured with normal cell medium, no significant differences in the OD values were observed, indicating that the prepared scaffolds exhibited no cytotoxicity. In summary, all scaffolds supported HUVEC and 293T cell adhesion and proliferation without cytotoxic effects, demonstrating excellent biocompatibility.

### 3.3. In Vitro AAV Release and Cell Transduction Assay

#### 3.3.1. Quantitative Analysis of AAV Release

**Figure 5:**
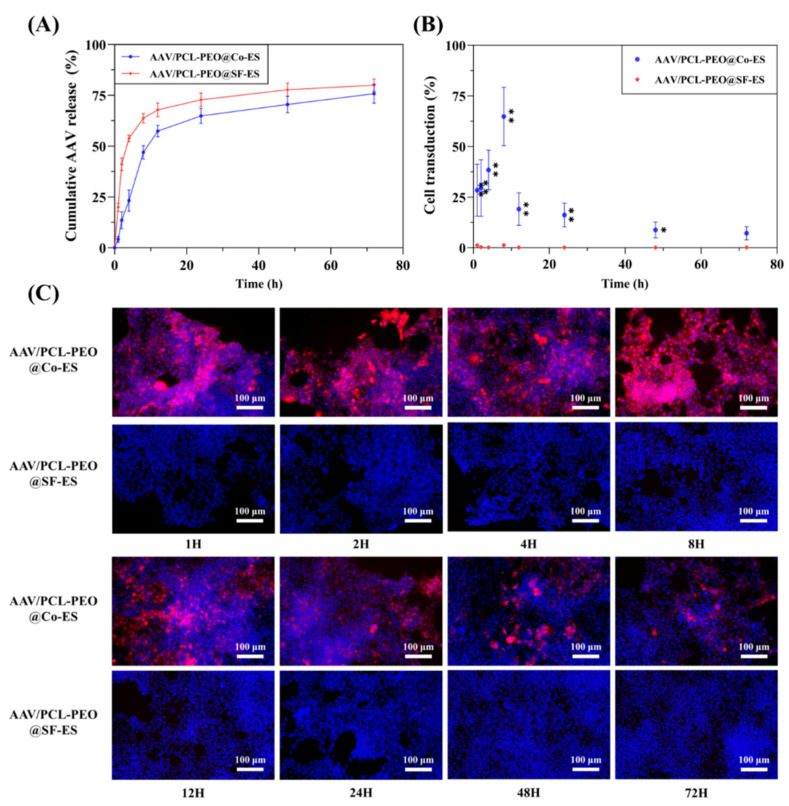
In vitro evaluation of AAV/PCL-PEO@SF-ES and AAV/PCL-PEO@Co-ES leaching solution. (**A**) AAV release from AAV/PCL-PEO@SF-ES and AAV/PCL-PEO@Co-ES scaffolds, as quantified by qPCR. (**B**) Percentage of mCherry-expressing cells relative to total cells. Ten random images were analyzed and the percentages of mCherry-positive cells were manually counted. (**C**) Fluorescent images of 293T cells incubated with the leachates from AAV/PCL-PEO@SF-ES and AAV/PCL-PEO@Co-ES containing AAV9-cmv-mCherry; blue: DAPI-stained nuclei, red: mCherry-expressing cells. Statistical significance was defined as * *p* < 0.05 and ** *p* < 0.01.

To evaluate the effectiveness of the prepared AAV-loaded scaffolds as viral vector delivery systems, an in vitro release test was conducted and the viral genome copies released at various time points were quantified using qPCR. Figure 5A illustrates the release profile of AAV from AAV/PCL-PEO@SF-ES and AAV/PCL-PEO@Co-ES scaffolds. AAV/PCL-PEO@SF-ES exhibited a rapid initial release, with approximately 53% of the AAV released within the first 4 h, followed by a slower release phase. This rapid initial release is likely attributed to the SF-ES technique [49,50], which results in AAV being distributed on or near the fiber surface, causing an initial burst release. The subsequent slow release of AAV is likely due to the dissolution of PEO in the scaffold upon contact with water, enabling the gradual release of the residual AAV. However, some studies have suggested that burst release of genes may result in the loss of activity in untransduced regions, thereby reducing overall transduction efficiency [20,51,52]. In this context, the high-dose release of AAV at the initial stages of delivery could contribute to a significant loss of activity and reduced overall therapeutic efficacy. This burst release may lead to localized overloads of viral particles, potentially reducing the precision and control of gene transfer, and raising concerns about toxicity and immune responses in vivo.

Compared to the AAV/PCL-PEO@SF-ES scaffold, the AAV/PCL-PEO@Co-ES scaffold exhibited a significantly slower release rate, with only 23% of AAV released within the first 4 h. This slower release is attributed to the core–shell fiber structure, where the outer PCL-PEO layer acts as a barrier, preventing the rapid diffusion of AAV from the core into the medium and effectively inhibiting the initial burst release. Over the next 24 h, AAVs released steadily increased to 65% as PEO dissolved and solvent activation facilitated release from the fibers. This gradual and controlled release mitigates the high-dose administration issues associated with burst release, thereby improving transduction efficiency and potentially reducing risks related to excessive local vector concentrations. In conclusion, the AAV/PCL-PEO@Co-ES scaffold, fabricated via Co-ES, exhibits excellent controlled release properties, effectively suppressing the initial burst release of AAV and avoid low transfection efficiency caused by rapid release, and minimizes AAV loss during delivery.

#### 3.3.2. Cell Transduction

To assess the cell transduction ability of AAV released from the AAV/PCL-PEO@SF-ES and AAV/PCL-PEO@Co-ES scaffolds, 293T cells were cultured with the scaffold leaching solution collected at various time points. Figure 5B,C shows the percentage of mCherry-positive cells and the transduction results, revealing that the cell transduction results for the AAV/PCL-PEO@Co-ES scaffold are consistent with the in vitro release trend. The limited AAV release during the first 4 h resulted in the transduction of approximately 35% of the cells. Over the subsequent 24 h, sustained AAV release from the scaffold led to continuous and stable transduction, with nearly 60% of the cells being transduced. Even at 72 h, nearly 10% of the cells still exhibited transduction. In contrast, the leaching solution from the AAV/PCL-PEO@SF-ES scaffold exhibited very low levels of cell transduction throughout the entire culture period. This is consistent with previous reports indicating that the SF-ES technique, which exposes viruses to an organic solvent environment during preparation, can compromise their stability, functionality, and structure, thereby reducing or entirely impairing their cell transduction ability [19,20,27]. These findings demonstrate that the AAV/PCL-PEO@Co-ES scaffold, constructed based on the Co-ES technique, not only effectively preserves the bioactivity of the vector but also enables a stable release profile and high gene transduction efficiency.

### 3.4. Histological Analysis

**Figure 6:**
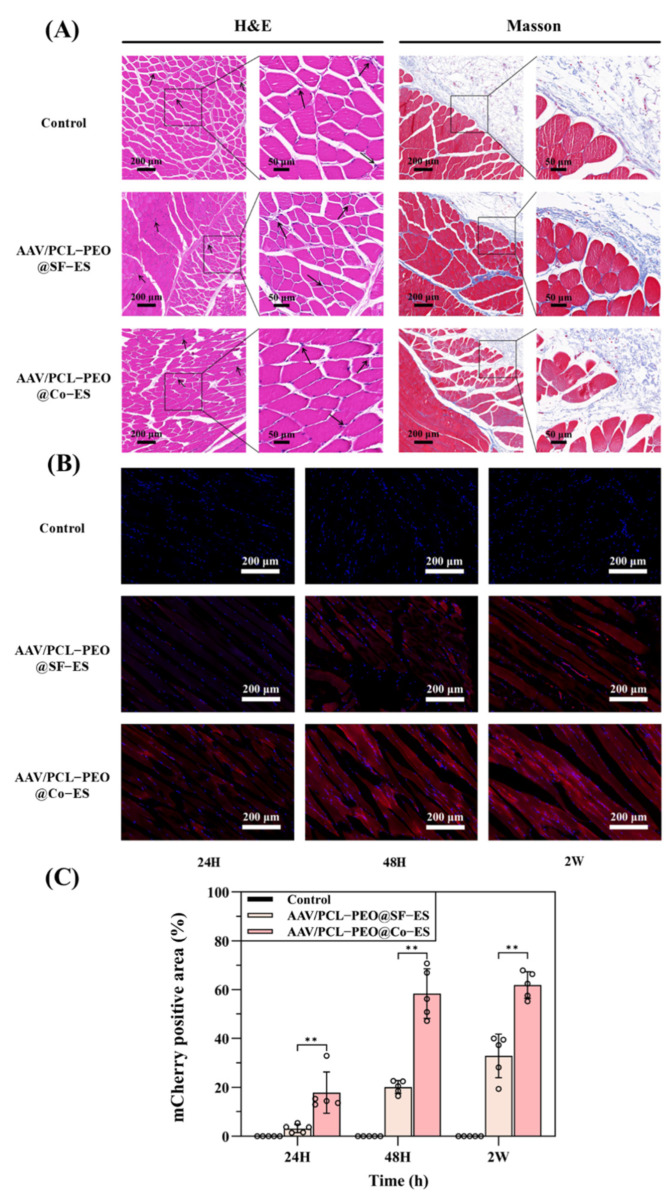
(**A**) H&E and Masson’s histological staining analysis of the blank control group, AAV/PCL-PEO@SF-ES group, and AAV/PCL-PEO@Co-ES group at 2 weeks; black arrow: inflammatory cells. (**B**) Fluorescence images of mCherry (red) expression at 2 weeks in the blank control group, AAV/PCL-PEO@SF-ES group, and AAV/PCL-PEO@Co-ES group, with DAPI-stained nuclei (blue). (**C**) Quantitative analysis of mCherry-positive areas. Error bars represent the standard deviation (*n* = 5), with statistical significance defined as ** *p* < 0.01. Circles in the figure represent individual data points used for statistical analysis.

To further evaluate the in vivo biocompatibility and viral delivery efficacy of the prepared AAV-loaded scaffolds, the scaffolds were implanted into SD rats and cultured for two weeks. Histological analysis was conducted on tissue sections using H&E staining, Masson staining, and fluorescence imaging. Figure 6A shows the H&E and Masson staining images at various magnifications for the blank control group, AAV/PCL-PEO@SF-ES group, and AAV/PCL-PEO@Co-ES group. H&E staining reveals a comparable number of inflammatory cells (black arrows) around the tissues in the experimental and control groups, with no evident signs of inflammation. Masson staining further illustrates the integration of tissue with the scaffolds [40,53]. As depicted in Figure 6A, collagen fibers (blue) are formed around the scaffolds in the experimental groups, indicating the excellent biocompatibility of the AAV/PCL-PEO@SF-ES and AAV/PCL-PEO@Co-ES scaffolds with surrounding tissues.

Furthermore, to evaluate the in vivo transduction efficiency of the AAV-loaded scaffolds, fluorescence imaging was performed to observe the mCherry fluorescence signal in skeletal muscle cells at various release intervals (24 h, 48 h, and 2 weeks) following a two-week uniform transduction period. As shown in Figure 6B, fluorescence imaging revealed that the cells, displaying characteristic skeletal muscle striations, exhibited a significantly stronger mCherry fluorescence signal at all time points in the experimental groups compared to the blank control group. Among the experimental groups, the AAV/PCL-PEO@Co-ES group suggested the largest mCherry-positive area, indicating the best transduction efficiency, which is consistent with the in vitro cell transduction results. Interestingly, although the AAV/PCL-PEO@SF-ES scaffold exhibited poor transduction efficiency in vitro, it still showed some transduction capability in vivo. This may be attributed to the fact that AAVs often exhibit reduced activity under in vitro conditions due to factors such as enzymatic degradation, limited ECM interactions, and suboptimal culture environments, whereas the in vivo environment provides more favorable conditions for viral stability and gene transfer [54].

The quantitative analysis in Figure 6C reveals that 24 h after in vivo release, the transduction area of the surrounding tissue around the AAV/PCL-PEO@Co-ES scaffold reached approximately 20%. The lower transduction efficiency observed within the first 24 h compared to in vitro cell experiments may be attributed to the homogeneous diffusion of AAV in the culture medium in vitro, which facilitates rapid diffusion, whereas the complex in vivo environment, including the viscosity and hydration of subcutaneous tissues, may slow down the diffusion rate. After 48 h, the transduction efficiency peaked with a transduction area of approximately 60%, remaining consistent with the results observed at 2 weeks. These in vivo results suggest that encapsulating AAVs within the core layer of the core–shell fibers in AAV/PCL-PEO@Co-ES scaffolds via Co-ES technology is an efficient method for viral vector delivery.

## 4. Conclusions

In this study, an AAV-loaded scaffold, AAV/PCL-PEO@Co-ES, was developed via Co-ES technology to achieve viral vector protection, sustained release, and enhanced biocompatibility. The PCL-PEO shell encapsulates AAVs within the fiber core, providing a physical barrier that preserves viral viability and effectively suppresses burst release. The scaffold exhibited excellent mechanical performance (tensile strength: 3.22 ± 0.48 MPa) and improved hydrophilicity (water contact angle: 67.90 ± 8.45°) due to the incorporation of PEO. In vitro AAV release and transduction studies demonstrated that the scaffold maintained viral bioactivity, effectively suppressed the initial burst release of AAV, and achieved sustained release. Furthermore, in vivo implantation further confirmed the biocompatibility of the scaffold and its feasibility for localized viral vector delivery. While the current work primarily focused on the temporal control of AAV release, the spatial distribution of gene transduction remains to be systematically investigated. Future work will aim to address this limitation by employing more complex tissue models and spatially resolved imaging techniques to evaluate region-specific delivery and ensure minimal off-target transduction. Overall, the AAV/PCL-PEO@Co-ES scaffold represents a promising platform for localized and sustained gene therapy, offering a potential strategy to overcome limitations associated with conventional viral vector administration, such as burst release and short expression duration.

## Data Availability

The original contributions presented in this study are included in the article. Further inquiries can be directed to the corresponding authors.

## Abbreviations

The following abbreviations are used in this manuscript:

AAV	adeno-associated virus
PCL	polycaprolactone
PEO	polyethylene oxide
WCA	water contact angle
ECM	extracellular matrix
SF-ES	single-fluid electrospinning
ELP	elastin-like polypeptide
Co-ES	coaxial electrospinning
PEUU	polyester urethane urea
PEEUU	polyester ether urethane urea
FBGC	foreign body giant cells
SD	Sprague Dawley
DCM	dichloromethane
DMF	N, N dimethylformamide
PBS	phosphate-buffered saline
SEM	scanning electron microscopy
TEM	transmission electron microscopy
FTIR	Fourier transform infrared spectrometer
HUVECs	human umbilical vein endothelial cells
DMEM	Dulbecco’s modified Eagle’s medium
FBS	fetal bovine serum
CCK-8	cell counting kit-8
H&E	hematoxylin and eosin
Masson	Masson’s trichrome
SD	standard deviation
ANOVA	analysis of variance

## References

[1] High K.A., Roncarolo M.G. (2019). Gene Therapy. N. Engl. J. Med..

[2] Pan X., Veroniaina H., Su N., Sha K., Jiang F., Wu Z., Qi X. (2021). Applications and developments of gene therapy drug delivery systems for genetic diseases. Asian J. Pharm. Sci..

[3] Quadir S.S., Choudhary D., Singh S., Choudhary D., Chen M.-H., Joshi G. (2024). Genetic frontiers: Exploring the latest strategies in gene delivery. J. Drug Deliv. Sci. Technol..

[4] Wang Y., Shao W. (2023). Innate Immune Response to Viral Vectors in Gene Therapy. Viruses.

[5] Atasoy-Zeybek A., Kose G.T., Turksen K. (2018). Gene Therapy Strategies in Bone Tissue Engineering and Current Clinical Applications. Cell Biology and Translational Medicine, Vol 4: Stem Cells and Cell Based Strategies in Regeneration.

[6] Wang J.-H., Gessler D.J., Zhan W., Gallagher T.L., Gao G. (2024). Adeno-associated virus as a delivery vector for gene therapy of human diseases. Signal Transduct. Target. Ther..

[7] Zhao Z., Anselmo A.C., Mitragotri S. (2022). Viral vector-based gene therapies in the clinic. Bioeng. Transl. Med..

[8] Li X., Le Y., Zhang Z., Nian X., Liu B., Yang X. (2023). Viral Vector-Based Gene Therapy. Int. J. Mol. Sci..

[9] Shirley J.L., de Jong Y.P., Terhorst C., Herzog R.W. (2020). Immune Responses to Viral Gene Therapy Vectors. Mol. Ther..

[10] Butt M.H., Zaman M., Ahmad A., Khan R., Mallhi T.H., Hasan M.M., Khan Y.H., Hafeez S., Massoud E.E.S., Rahman M.H. (2022). Appraisal for the Potential of Viral and Nonviral Vectors in Gene Therapy: A Review. Genes.

[11] Rincon M.Y., VandenDriessche T., Chuah M.K. (2015). Gene therapy for cardiovascular disease: Advances in vector development, targeting, and delivery for clinical translation. Cardiovasc. Res..

[12] van der Meel R., Vehmeijer L.J.C., Kok R.J., Storm G., van Gaal E.V.B. (2013). Ligand-targeted particulate nanomedicines undergoing clinical evaluation: Current status. Adv. Drug Deliv. Rev..

[13] Selkirk S.M. (2004). Gene therapy in clinical medicine. Postgrad. Med. J..

[14] Wang Y., Hu J.K., Krol A., Li Y.P., Li C.Y., Yuan F. (2003). Systemic dissemination of viral vectors during intratumoral injection. Mol. Cancer Ther..

[15] Hadjizadeh A., Ghasemkhah F., Ghasemzaie N. (2017). Polymeric Scaffold Based Gene Delivery Strategies to Improve Angiogenesis in Tissue Engineering: A Review. Polym. Rev..

[16] Wang Y., Bruggeman K.F., Franks S., Gautam V., Hodgetts S.I., Harvey A.R., Williams R.J., Nisbet D.R. (2021). Is Viral Vector Gene Delivery More Effective Using Biomaterials?. Adv. Healthc. Mater..

[17] Beer S.J., Hilfinger J.M., Davidson B.L. (1997). Extended release of adenovirus from polymer microspheres: Potential use in gene therapy for brain tumors. Adv. Drug Deliv. Rev..

[18] Kidd M.E., Shin S., Shea L.D. (2012). Fibrin hydrogels for lentiviral gene delivery in vitro and in vivo. J. Control. Release.

[19] Puhl D.L., Mohanraj D., Nelson D.W., Gilbert R.J. (2022). Designing electrospun fiber platforms for efficient delivery of genetic material and genome editing tools. Adv. Drug Deliv. Rev..

[20] Ji W., Sun Y., Yang F., van den Beucken J.J.J.P., Fan M., Chen Z., Jansen J.A. (2011). Bioactive Electrospun Scaffolds Delivering Growth Factors and Genes for Tissue Engineering Applications. Pharm. Res..

[21] Lee S., Jin G., Jang J.-H. (2014). Electrospun nanofibers as versatile interfaces for efficient gene delivery. J. Biol. Eng..

[22] Zhang N., Milbreta U., Chin J.S., Pinese C., Lin J., Shirahama H., Jiang W., Liu H., Mi R., Hoke A. (2022). Biomimicking Fiber Scaffold as an Effective In Vitro and In Vivo MicroRNA Screening Platform for Directing Tissue Regeneration (vol 6, 1800808, 2019). Adv. Sci..

[23] Zulkifli M.Z.A., Nordin D., Shaari N., Kamarudin S.K. (2023). Overview of Electrospinning for Tissue Engineering Applications. Polymers.

[24] Furuno K., Suzuki K., Sakai S. (2024). Transduction and Genome Editing of the Heart with Adeno-Associated Viral Vectors Loaded onto Electrospun Polydioxanone Nonwoven Fabrics. Biomolecules.

[25] Kim J., Son Y.-W., Hwang K., Park H.-W., Kim Y., Kim M., Shin J.E., Park K.I., Lee S., Jang J.-H. (2023). Synergistic Enhancement of Adeno-Associated Virus-Mediated In Vivo Direct Neuronal Reprogramming by Spatially Aligned Fibrous Matrices in Spinal Cord Injury Models. Adv. Ther..

[26] Lee S., Kim J.-S., Chu H.S., Kim G.-W., Won J.-I., Jang J.-H. (2011). Electrospun nanofibrous scaffolds for controlled release of adeno-associated viral vectors. Acta Biomater..

[27] Gu X., Matsumura Y., Tang Y., Roy S., Hoff R., Wang B., Wagner W.R. (2017). Sustained viral gene delivery from a micro-fibrous, elastomeric cardiac patch to the ischemic rat heart. Biomaterials.

[28] Sharon D., Kamen A. (2018). Advancements in the design and scalable production of viral gene transfer vectors. Biotechnol. Bioeng..

[29] Nayerossadat N., Maedeh T., Ali P.A. (2012). Viral and nonviral delivery systems for gene delivery. Adv. Biomed. Res..

[30] Pupo A., Fernandez A., Low S.H., Francois A., Suarez-Amaran L., Samulski R.J. (2022). AAV vectors: The Rubik’s cube of human gene therapy. Mol. Ther..

[31] Hezi-Yamit A., Sullivan C., Wong J., David L., Chen M., Cheng P., Shumaker D., Wilcox J.N., Udipi K. (2009). Impact of polymer hydrophilicity on biocompatibility: Implication for DES polymer design. J. Biomed. Mater. Res. Part A.

[32] Jones J.A., Chang D.T., Meyerson H., Colton E., Kwon I.K., Matsuda T., Anderson J.M. (2007). Proteomic analysis and quantification of cytokines and chemokines from biomaterial surface-adherent macrophages and foreign body giant cells. J. Biomed. Mater. Res. Part A.

[33] Mitxelena-Iribarren O., Riera-Pons M., Pereira S., Jose Calero-Castro F., Castillo Tunon J.M., Padillo-Ruiz J., Mujika M., Arana S. (2023). Drug-loaded PCL electrospun nanofibers as anti-pancreatic cancer drug delivery systems. Polym. Bull..

[34] Song Y., Hu Q., Liu S., Yao G., Zhang H. (2024). Electrospinning drug-loaded polycaprolactone/polycaprolactone-gelatin multi-functional bilayer nanofibers composite scaffold for postoperative wound healing of cutaneous squamous cell carcinoma. Biomed. Technol..

[35] Garg S.M., Paiva I.M., Vakili M.R., Soudy R., Agopsowicz K., Soleimani A.H., Hitt M., Kaur K., Lavasanifar A. (2017). Traceable PEO-poly(ester) micelles for breast cancer targeting: The effect of core structure and targeting peptide on micellar tumor accumulation. Biomaterials.

[36] Asghari F., Faradonbeh D.R., Malekshahi Z.V., Nekounam H., Ghaemi B., Yousefpoor Y., Ghanbari H., Faridi-Majidi R. (2022). Hybrid PCL/chitosan-PEO nanofibrous scaffolds incorporated with *A. euchroma* extract for skin tissue engineering application. Carbohydr. Polym..

[37] Hu Q., Tang W., Song Y., Zhang H. (2025). Preparation and characterization of drug-loaded coaxial electrospun nanofibers membranes with pH-responsive and antibacterial properties. J. Drug Deliv. Sci. Technol..

[38] Hu Q., Zhang Y., Song Y., Shi H., Yang D., Zhang H., Gu Y. (2024). 3D printing/electrospinning of a bilayered composite patch with antibacterial and antiadhesive properties for repairing abdominal wall defects. J. Mater. Chem. B.

[39] Song Y., Hu Q., Liu S., Wang Y., Jia L., Hu X., Huang C., Zhang H. (2024). 3D printed biomimetic composite scaffolds with sequential releasing of copper ions and dexamethasone for cascade regulation of angiogenesis and osteogenesis. Chem. Eng. J..

[40] Song Y., Hu Q., Liu S., Wang Y., Zhang H., Chen J., Yao G. (2024). Electrospinning/3D printing drug-loaded antibacterial polycaprolactone nanofiber/sodium alginate-gelatin hydrogel bilayer scaffold for skin wound repair. Int. J. Biol. Macromol..

[41] Ahani E., Mianehro A. (2023). Electrospun scaffold based on poly(ethylene oxide)/poly(ε-caprolactone) for prolonged intravaginal antiviral drug release. J. Drug Deliv. Sci. Technol..

[42] Kupka V., Dvorakova E., Manakhov A., Michlicek M., Petrus J., Vojtova L., Zajickova L. (2020). Well-Blended PCL/PEO Electrospun Nanofibers with Functional Properties Enhanced by Plasma Processing. Polymers.

[43] Han Y., Xu Y., Zhang S., Li T., Ramakrishna S., Liu Y. (2020). Progress of Improving Mechanical Strength of Electrospun Nanofibrous Membranes. Macromol. Mater. Eng..

[44] Yang Y., Hu H. (2017). Spacer fabric-based exuding wound dressing—Part II: Comparison with commercial wound dressings. Text. Res. J..

[45] Hu Q., Wu J., Zhang H., Dong W., Gu Y., Liu S. (2022). Designing Double-Layer Multimaterial Composite Patch Scaffold with Adhesion Resistance for Hernia Repair. Macromol. Biosci..

[46] Abadi B., Goshtasbi N., Bolourian S., Tahsili J., Adeli-Sardou M., Forootanfar H. (2022). Electrospun hybrid nanofibers: Fabrication, characterization, and biomedical applications. Front. Bioeng. Biotechnol..

[47] Mondesert H., Bossard F., Favier D. (2021). Anisotropic electrospun honeycomb polycaprolactone scaffolds: Elaboration, morphological and mechanical properties. J. Mech. Behav. Biomed. Mater..

[48] Song Y.T., Hu Q.X., Liu Q., Liu S.H., Wang Y.H., Zhang H.G. (2023). Design and fabrication of drug-loaded alginate/hydroxyapatite/collagen composite scaffolds for repairing infected bone defects. J. Mater. Sci..

[49] Steyaert I., Van der Schueren L., Rahier H., de Clerck K. (2012). An Alternative Solvent System for Blend Electrospinning of Polycaprolactone/Chitosan Nanofibres. Macromol. Symp..

[50] Schoolaert E., Steyaert I., Vancoillie G., Geltmeyer J., Lava K., Hoogenboom R., De Clerck K. (2016). Blend electrospinning of dye-functionalized chitosan and poly(ε-caprolactone): Towards biocompatible pH-sensors. J. Mater. Chem. B.

[51] Sun K., Lin H., Tang Y., Xiang S., Xue J., Yin W., Tan J., Peng H., Alexander P.G., Tuan R.S. (2020). Injectable *BMP-2* gene-activated scaffold for the repair of cranial bone defect in mice. Stem Cells Transl. Med..

[52] Nie H., Wang C.-H. (2007). Fabrication and characterization of PLGA/HAp scaffolds for delivery of BMP-2 plasmid composite DNA. J. Control. Release.

[53] Hu X., Hu Q., Liu S., Zhang H. (2024). Synergy of engineered gelatin methacrylate-based porous microspheres and multicellular assembly to promote osteogenesis and angiogenesis in bone tissue reconstruction. Int. J. Biol. Macromol..

[54] Go N., Ahn C., Lee J.Y. (2024). Enhancing transduction efficiency of adeno-associated virus 9 by cell line engineering: Implication for gene therapy potency assay. Biotechnol. Bioprocess. Eng..

